# How Ought We to Treat Gonorrhea Epididymitis

**Published:** 1894-08

**Authors:** 


HOW OUGHT WE TO TREAT GONORRHEAL EPIDIDYMITIS? Dr. Henri Picard (*La France Medicale*) answers this question by stating that there is, or has been, constipation for a few days. This requires a purgative to free the digestive canal, and will relieve the compression on the rectum, the prostate and the urethra. The drastics, and especially aloes, should not be ordered, but the salines or castor oil. If the constipation continues, use large enemata.

Rest, in the horizontal position, is the next point to attend to. Along with this a good support must be fitted to the testicle. This affords a great deal of relief; and permits the patient to turn in bed without pain, and in subacute cases, to move around the room. Horand's support is the best. This support is made of a large piece of lozenge shaped linen, split at the lateral angles, and lined with a thick layer of cotton wool, placed on vulcanized linen. By means of the angles this support can be fastened to the body so as to hold the scrotum and testicles, and afford gentle pressure.

Demulcent drinks are of much value. They soothe the inflamed urethra, and render the urine less irritating. Linseed tea, infusion of violets, orange water, etc., are very good forms of drinks, and should be taken in large quantities.

A bath once or twice a day, or every other day, of the temperature of 95° F. is useful. These baths require to be prolonged. There is not much benefit from them unless kept up for some time. They calm the pain and favor sleep.

For the pain and tenderness the best remedy is salicylate of soda in doses of three to four grammes daily. Antipyrine has been used with advantage, and the anemone pulsatilla has been found very beneficial in allaying pain and swelling.

When there is much swelling, redness of the skin, and the scrotum and testicles are very painful and tender, half a dozen leeches should be applied along the cord. If the condition of the tissues is serious, even a dozen may be used.

Later on, iodide of potash ought to be employed to remove the induration and enlargement that has been left by the disease. Mercurial strapping or ointment may also be employed for these conditions. In spite of all efforts it is sometimes impossible to restore the testicle and epididymis to their normal size. The vas deferens is sometimes obliterated, and the seminal fluid cannot make its way from the corresponding testicle.—*Med. and Surg. Reporter.*

